# ChatGPT as a mental health advisory service: Comparing evaluations from youth and health professionals

**DOI:** 10.1177/20552076261427447

**Published:** 2026-02-26

**Authors:** Marita Skjuve, Asbjørn Følstad, Petter Bae Brandtzaeg

**Affiliations:** 1 555969 SINTEF Digital, Oslo, Norway; 2 Department of Media and Communication, 6305 University of Oslo, Oslo, Norway

**Keywords:** ChatGPT, artificial intelligence, LLM, professional evaluation, hybrid advisory models, youth mental health

## Abstract

**Objective:**

Despite the growing use of artificial intelligence in youth mental health support, little is known about how either young people or health professionals perceive answers to mental health-related inquiries generated by large language models (LLMs). Therefore, we draw on Media Richness Theory to examine how these two user groups perceive the “richness” of text-based communication in this context and whether young people and health professionals differ in their assessment.

**Methods:**

A total of 123 young people and 31 health professionals evaluated answers to youth mental health inquiries. Each inquiry had two blinded answers: one generated by ChatGPT (GPT-4) and one written by a health professional. Participants rated the answers for validation, relevance, clarity, and utility and were asked to recommend one or both answers. Open-ended responses elaborating participant choices were also collected.

**Results:**

The quantitative findings show that young people and health professionals rated answers from both sources similarly on validation, clarity, and utility. However, young people rated ChatGPT's answers higher for relevance and utility, finding them “richer.” This was supported by qualitative data, where youth preferred ChatGPT's clear and actionable answers. Health professionals showed no strong preference and were more critical, often finding the answers too detailed or lacking empathy.

**Conclusion:**

This study is the first to compare youth and professional perspectives on ChatGPT's role in youth mental health advice within a blind evaluation design. We conclude by proposing a hybrid advisory model that combines professional expertise with LLMs to enhance the efficiency, scale, and accessibility of youth mental health advisory services.

## Introduction

The integration of artificial intelligence (AI) into mental health services is rapidly offering new ways to provide information about mental health concerns.^1^ Large language models (LLMs) such as ChatGPT have demonstrated the potential to provide immediate answers to a wide range of inquiries,^2^ including mental health.^3,4^ Studies indicate that ChatGPT can potentially improve therapeutic processes^5^ and deliver supportive, empathetic responses.^6^ This has led to growing interest in using LLMs as complementary tools to support health professionals in delivering mental-health information and advice to young people. However, LLMs currently lack the adaptive sensitivity to linguistic nuances and contextual cues that humans demonstrate,^6^ a potentially critical limitation in text-based mental-health communication.

This makes determining whether and how LLMs can complement traditional mental health advisory services a critical research priority,^7^ particularly given the challenges associated with addressing youth mental health needs.^8^ Given the mixed findings and the potential role of LLMs in mental health support, it is pertinent to compare how young people (users) and health professionals (providers) perceive LLM-generated answers.

Young people require social support guidance that is accurate, empathetic,^9^ relatable, and appropriate to their developmental stage.^10^ They are often reluctant to seek professional help due to stigma, fear of judgment, or accessibility concerns,^11^ and may be drawn to available, seemingly anonymous LLM-based services. Health professionals are trained to deliver more nuanced support: despite their advantages, LLMs lack lived experience and intrinsic empathy. Moreover, the legitimacy of using LLMs in mental-health advisory services depends on public and professional acceptance and endorsement. Comparing these groups is essential to evaluate whether AI-generated answers align with professional ethics and safety standards while still meeting the informational and emotional needs of youth. Such a comparison represents a critical gap in the extant literature.

Our study addresses this gap by using a blind-evaluation design, enabling the first direct comparison of youth and professional assessments of ChatGPT's (GPT-4) mental-health guidance. This allows for a thorough investigation of the following research question:

How do young people and health professionals differ in their evaluations of ChatGPT's answers to mental health inquiries compared to answers written by health professionals?

Responding to this question, we apply Media Richness Theory to examine how young people and health professionals experience answers to youth mental health questions produced by different sources, namely, ChatGPT or health professionals. In total, 123 youth participants and 31 professional health participants evaluated these answers using qualitative and quantitative methods. As such, this study enhances our understanding of how LLMs can communicate mental-health guidance to young people compared to human professionals. Specifically, our findings show that both groups perceived ChatGPT to generate well-formulated, empathetic responses with actionable advice. However, youth participants found these qualities particularly convincing and rated ChatGPT's answers more positively overall, suggesting that they were more strongly influenced by its clear structure and supportive tone than the professional participants. We propose a hybrid human–AI advisory model that would see AI-driven support complement human expertise. Our study also contributes to theoretical discussions on the perceived richness and potential benefits of human–AI communication in the context of mental health.

## Background

Our research is conducted in a rapidly evolving field. To clearly position our research relative to existing work, this section details relevant background existing research on youth and online mental health advice, as well as young people's perceptions of AI-generated advice in general.

### Youth and mental health advice: From social media to LLMs?

Young people use the Internet to a large extent when seeking information about their mental health.^8^ Social media platforms have become a particularly attractive channel for mental health-related information.^12,13^ According to Basch et al.,^14^ TikTok videos with a mental health-related focus can receive over 1 billion views. However, although the Internet and social media provide easy access to information, they are also polluted by health misinformation.^15^ Furthermore, the lack of information tailored to the needs of young people struggling with mental health issues remains a significant challenge.^16^

Research has aimed to address these challenges by developing methods to provide young people with accessible, high-quality, and judgment-free information,^8^ with LLMs receiving significant attention as a potential solution.^17^ One study has even suggested that young people will increasingly depend less on human interactions and rely more on LLM-based solutions for tailored information, relational experiences, practical assistance, and emotional support.^18^

A key reason for this trend is that LLMs are designed to simulate human-like interactions, delivering personalized and rapid responses around the clock. Recent advancements have made LLMs increasingly sophisticated, enabling more nuanced, context-aware, and adaptive interactions with a wide knowledge base and allowing them to address most inquiry types at any time.^19^ Furthermore, research indicates that ChatGPT may display empathy^20^ and emotional awareness.^21^ This transformative potential in LLMs extends across industries ranging from customer service^22^ and education^23^ to advisory roles and healthcare,^24^ including mental health.^25^ As such, LLMs such as ChatGPT are seen to hold the potential to offer young people low-threshold access to mental health information and to support health professionals working in online help services by reducing repetitive tasks and increasing efficiency.^26^ For instance, Gupta et al.^27^ explored how young people (18–25 years) view LLMs such as ChatGPT. Half of the participants used LLMs frequently, and around 40% were comfortable using such AI for social support. However, the participants expressed concerns about trust and privacy. Elsewhere, Mármol-Romero et al.^28^ developed an AI solution to provide mental health support for adolescents, finding that the participants perceived the AI as capable of giving good advice. Adopting a different approach, Johnson et al.^29^ used an LLM as part of an intervention to reduce perfectionism in young people. Their participants reported that the AI-generated advice provided valuable information but also indicated that they perceived that advice as impersonal.

Although LLMs have demonstrated potential in mental health contexts, existing research indicates a dichotomy in perceptions of LLM- or AI-based advisory tools. Some young people value the consistency and nonjudgmental nature of AI-generated responses^30^; others critique their lack of empathy and contextual depth.^31^ Furthermore, substantial concern has been voiced regarding potential challenges related to the use of LLMs for mental health purposes. For example, LLMs that overly agree with users—behaving in a sycophantic manner—may fuel misconceptions or delusions.^32^ Furthermore, the inherent risk of LLM hallucinations and misinformation may be critical in the context of mental health.^1^ In mental health contexts, where trustworthiness, empathy, and information quality are critical, these deficiencies can undermine the perceived capabilities of LLMs. This makes it crucial to evaluate whether AI solutions powered by LLMs meet the needs of young people in terms of informational accuracy and emotional support.^33^

### Perceptions of AI-generated advice

Several studies have explored how people perceive advice generated by AI compared to advice produced by human professionals, particularly in the medical domain.^34,35,36,37^ Ayers et al.^35^ found that medical professionals preferred AI-generated medical advice over the advice of doctors, stating that the AI-generated output provided higher-quality advice and appeared more empathetic. Similarly, Singhal et al.^36^ found that answers to medical questions generated by AI were preferred over those provided by a generalist physician but not over answers produced by specialists. Kim et al.^38^ compared ChatGPT's ability to generate answers to sleep-related questions with answers provided by sleep specialists. They found that laypersons rated ChatGPT as more helpful and empathic, while professionals rated ChatGPT higher on empathy but found the specialists’ answers more helpful.

Elsewhere, Vowels^39^ explored ChatGPT's ability to provide relationship counseling. They found that participants rated ChatGPT's answers as more helpful and empathetic than those provided by relationship experts. This assessment was further validated by professional therapists who judged the AI-generated answers helpful and empathetic. Similarly, a study by Hatch et al.^5^ suggested that ChatGPT can generate therapeutic responses often indistinguishable from those written by professional therapists. Here, AI-generated responses were also rated higher in key psychotherapy principles.

Notably, most studies have focused on nonyouth populations when comparing AI-generated output to that produced by human experts. We are aware of only one study that specifically addresses young people: Young et al.^17^ explored how young people rate advice given by peers, adult mentors, therapists, and AI on questions related to relationships, suicidal thoughts, self-expression, and physical health. They found that participants tended to prefer advice from the adult mentor, followed by AI-generated advice. Moreover, preferences for AI output appeared to be context- or topic-sensitive. When questions concerned suicidal thoughts, advice from an adult mentor was preferred, while AI-generated responses were favored for less sensitive topics.

Although studies indicate that LLMs and AI-generated answers may be perceived as helpful across various domains, knowledge of how young people evaluate ChatGPT's responses to mental health questions compared to those of professionals remains scarce. Some studies suggest that different groups of people, such as laypersons and experts, assess AI-generated answers differently (e.g., Vowels^39^; Singhal et al.^36^; Kim et al.^38^). However, they do not discuss how or why such differences exist. It is likely that young people and health professionals approach answers to mental health questions from different perspectives. Youth participants offer valuable insights into how AI-generated content meets their needs and resonates with their experiences. In contrast, health professionals can evaluate their adherence to clinical guidelines and ethical standards.

## Theoretical approach

Media Richness Theory^40^ provides a framework for evaluating communication effectiveness based on a medium's ability to reduce ambiguity and facilitate understanding. Given that mental health discussions often involve sensitive, emotionally charged, and complex issues, analyzing richness appears crucial.

According to Daft et al.,^41^ media richness is determined by factors such as the ability to provide immediate feedback (e.g., the possibility for follow-up questions), convey multiple cues (e.g., tone and empathy), language variety (e.g., the use of emojis, numbers, and other symbols), and personal focus (e.g., messages tailored to the individual). Face-to-face interactions are considered high in media richness because they can convey multiple cues, provide immediate feedback, and personalize communication.^41^ In contrast, a written letter is generally regarded as low in media richness due to its lack of immediacy and limited nonverbal cues. However, the richness of written communication can vary depending on tone, empathy, and language variety, factors that influence how effectively written communication conveys its message.

The demand for rich media experiences and the extent to which they relate to AI-generated content depend on the need to reduce uncertainty or ambiguity (equivocality). In youth mental health support, where trust is key, media richness may shape user experiences. Research has shown that richer communication media enhance the perceived effectiveness of social support and information in healthcare settings.^42,43^ Meanwhile, studies suggest that the optimal degree of media richness in interactions with AI-generated communication varies by context, with some interactions requiring higher degrees of immediacy, empathy, and adaptability than others.^17^

Applying the Media Richness Theory allows us to assess whether LLM-generated mental health advice meets users’ informational and emotional needs. We evaluate perceived validation, relevance, clarity, and utility to assess whether participants experience greater richness, and then analyze their written reflections on LLM-generated versus human-provided support. These two steps contribute to understanding how LLM-generated answers compare to answers written by health professionals, and whether the answers align with the media richness needs of different mental health inquiries.

## Method

To ensure transparency, details about the study, including the methods, survey questionnaire, and ethical considerations, are available on the Open Science Framework (OSF) (https://osf.io/x7cw9).

**Figure 1.**
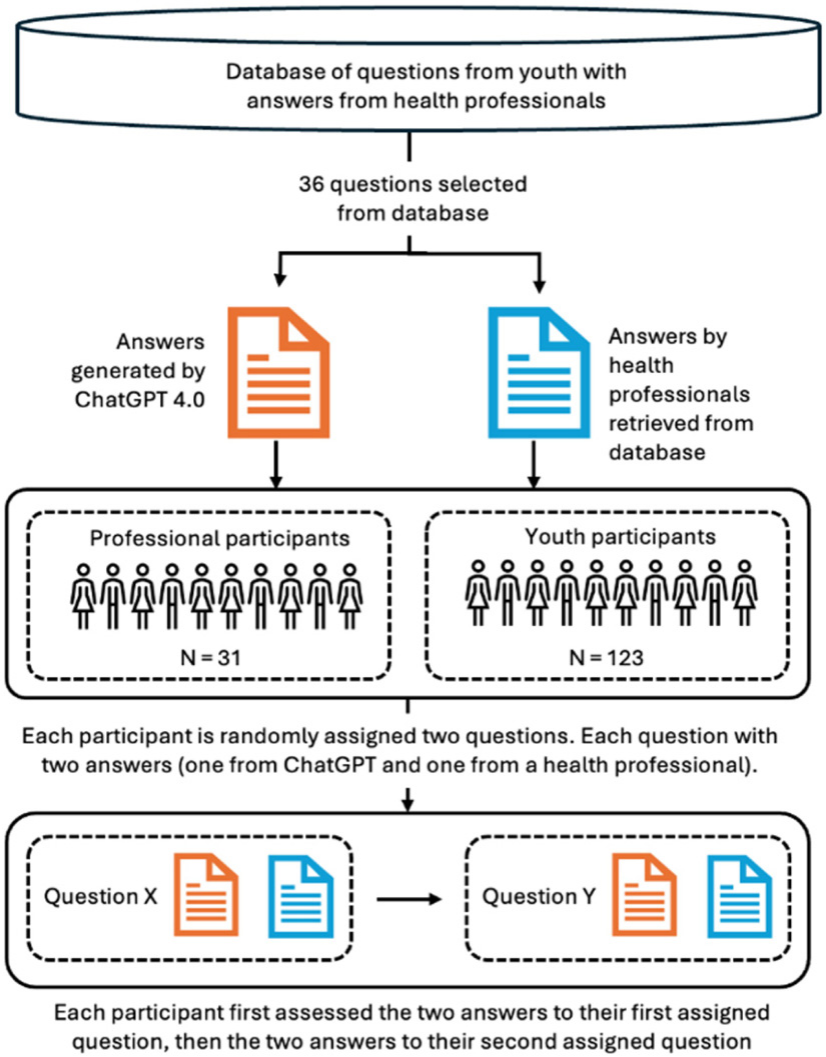
Overview of the data collection procedure.

We used a mixed-methods design to investigate how two groups—young people and health professionals—perceived answers to youth mental health inquiries produced by ChatGPT compared to responses provided by health professionals. The latter were collected from www.ung.no, a Norwegian governmental question-and-answer service for young people aged 13–20, referred to as the “database” in Figure 1. On this platform, young people can anonymously submit questions that are answered by professionals such as psychologists, social workers, doctors, and other health personnel. We investigated these two groups because young people are the primary recipients of such mental health advice, while health professionals may provide expert evaluations, allowing for a comprehensive comparison of perceptions of AI-generated and human-authored answers.

Participants in the two groups were presented with identical questions taken from the www.ung.no platform. With each question, participants were presented with one ChatGPT answer and one from a health professional. Answers were blinded to eliminate bias, ensuring that the participants were unaware of the source of the answer. The participants evaluated these answers independently using structured criteria. The data collection process is described in detail below and illustrated in Figure 1.

### Sample

The study involved two participant groups: “youth participants” and “professional health participants.” It should be noted that the young people and health professionals who provided the original questions (youth) and original responses (health professionals) taken from the database (ung.no) are not categorized as “participants,” as Figure 1 illustrates.

#### Group 1: Youth participants

We recruited 123 youth participants (98 females, 17 males, and eight nonbinary or unspecified) aged 16–20, with a median age of 17, from six high schools across urban and rural areas in Norway. Most had experience with ChatGPT or MyAI, two LLM-based services popular among young people: 9% reported using ChatGPT or MyAI several times a day, 5% daily, 32% weekly, and 41% rarely; 14% had never used ChatGPT or MyAI.

Data collection occurred in classrooms during school hours in the fall of 2023, with each session lasting 45 min. Schools were selected to represent different geographic locations, academic and vocational tracks, and sociocultural backgrounds to ensure diverse perspectives. The study aimed to capture a broad understanding of youth perspectives on mental health advisory services, recognizing the influence of individual, social, and cultural factors.^10^ Therefore, the sample size was determined pragmatically, based on the feasibility of school recruitment and the goal of achieving broad representation across school types and sociocultural contexts.

#### Group 2: Professional health participants

The study's 31 trained professional health participants included psychologists, social workers, and educators at www.ung.no. As active contributors to ung.no, these participants had direct experience with how such advisory systems should respond to youth with mental health issues. This group was recruited via email by their coordinator in October 2023. Participation was limited by the size of the available professional population (*N* = 40), of which 31 agreed to participate. The average age was 49 (range = 31–65 years). Twenty-seven identified as female, two as male, and two did not report gender. 10% used ChatGPT or MyAI weekly, 30% used it rarely, and 60% had never used either service. The participants received the study invitation and questionnaire by email.

### Procedure

*Question selection.* The study used a set of 36 anonymized questions (see OSF) selected from a database of 324 mental health-related questions and answers provided by www.ung.no (Figure 1). Question selection was based on specific criteria:

- Concerns about common mental health issues relevant to youth aged 16–20.
- Exclusion of highly sensitive topics such as eating disorders, sexual abuse, and suicidal thoughts to protect youth participants.
- Removal of questions with highly specific information, such as details about the questioner's location or parents’ occupation.
- Selection of questions postdating September 2021 to ensure they were not part of ChatGPT's training data at the time of the study.
- Representation of a balanced mix of genders and topics to reflect the diverse concerns of youth.

*Answer generation.* Two answers accompanied each mental health question: one generated by ChatGPT and one authored by human health professionals at www.ung.no. We used ChatGPT (GPT-4 model) to generate answers, ensuring a consistent, up-to-date, and high-performing AI model for comparison with human-authored answers, enabling a robust evaluation of AI-generated mental health advice. These answers were generated in May 2023 with no additional instructions beyond the original question text, to ensure consistency. We performed context cleaning between generated answers to avoid contamination across sessions. The answers were generated in Norway and in Norwegian.

*Blind test.* To ensure unbiased evaluations, participants were unaware of the source of each answer. We excluded any identifiable information, such as disclaimers specific to ChatGPT (e.g., “As an AI, I cannot provide a diagnosis”) and references to the platform (e.g., “Thanks for reaching out to ung.no”). Several answers authored by health professionals inadvertently included references to ung.no, but these instances were minimal.

*Saturation in analysis.* Given that each participant provided two open-ended evaluations, resulting in a substantial corpus of qualitative material, we ascertained that saturation would be achieved during analysis. During coding, no new themes emerged after reviewing approximately two-thirds of the dataset, indicating saturation in analysis.

*Independent assessment.* Each participant was randomly assigned two mental health questions, each with two associated answers for assessment: one authored by a health professional and one generated by ChatGPT. Hence, each participant assessed four answers in total.

The participants were asked to evaluate each answer on four key criteria: validation, relevance, clarity, and utility.

- Validation: whether the answer acknowledges the user's feelings or concerns (e.g., showing empathy or understanding).
- Relevance: how well the answer addresses the specific issue raised in the question.
- Clarity: whether the answer is easy to understand and free of ambiguity.
- Utility: the extent to which the answer provides actionable or helpful guidance.

Each criterion was rated on a 7-point Likert scale from strongly disagree to strongly agree, adapted from the Session Rating Scale developed by Duncan et al.^44^ For each pair of answers, participants were also asked to recommend one or both answers and to explain, in an open-ended report, why they chose one over the other or both. We used identical questions and procedures for both participant groups to enable direct comparison between them. Participant responses were gathered through LimeSurvey. The questionnaire was piloted prior to the data collection.

### Ethical considerations

Participation was voluntary and anonymous, and IP addresses and personal data were not collected. To ensure ethical integrity, only nonsensitive questions were included to avoid provoking strong reactions in the participants. The participants consented electronically.

### Analysis

*Quantitative data.* Likert-scale ratings were analyzed using descriptive and inferential statistics. The Mann–Whitney *U* test was used to observe how young people and professional health participants evaluated answers produced by ChatGPT compared with those provided by health professionals. A Wilcoxon Test was conducted to compare participants’ evaluations of ChatGPT-generated answers versus those authored by health professionals across the four dimensions. To control for Type I error inflation due to multiple comparisons, Bonferroni corrections were applied to the *p*-values of all Mann–Whitney *U* and Wilcoxon tests involving the four evaluation variables. Specifically, four separate comparisons were conducted for each test set (Validation, Relevance, Clarity, Utility), and the adjusted significance level was therefore set to α = .0125.

Finally, we used a chi-square test to examine whether the participants recommended one answer over the other. The statistical analysis was performed using SPSS v30. All tests were conducted using the aggregated scores for each participant across both assessments (averages for Likert scale measures, combined scoring for preference scores). For quality assurance, we also performed separate analyses on each individual assessment, and the results from these analyses overlapped those based on the aggregated scores. Thus, this paper reports only aggregated scores.

*Qualitative data.* Following Braun and Clarke,^45,46^ we conducted thematic analysis of the qualitative data in the open-ended reports. First, we read through the reports to gain an overall understanding of the data. Next, one of the authors assigned codes to the reports, then merged the codes into higher-level themes. Finally, themes were reviewed and named, and illustrative quotes from participants were selected. Here, we followed Yeo and Han^47^ recommendations and chose quotes that would substantiate each theme. We also made sure to include quotes from multiple participants and did not edit them beyond translation from Norwegian to English.

The quality of the thematic analysis was ensured by a thorough review process, which saw another author examine all proposed codes and themes and suggest modifications when necessary. Disagreements occurred for 11% of the codes and were resolved through discussion. A chi-square test was performed to explore differences in perceptions between the two participant groups.

## Results

### Quantitative analysis results

#### Differences between participant groups in their evaluation of answers

**Figure 2.**
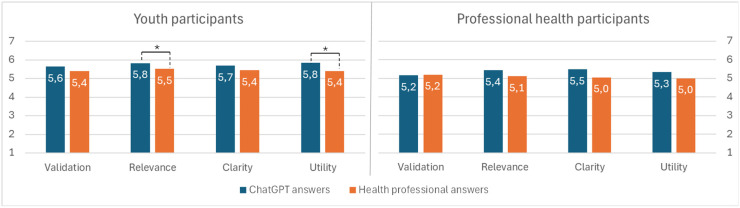
Participation groups and their different assessments of ChatGPT-generated answers vs. answers written by health professionals.

Here, we investigate differences between the participant groups. Figure 2 shows significant differences in how young people and professional health participants evaluated the answers across the four variables.

For ChatGPT-generated answers, youth participants assigned all four variables nominally higher scores than professional health participants. Before correction, differences were significant for *Validation* (*U* = 1450.5, *p* = .037) and *Utility* (*U* = 1364, *p* = .013). However, after Bonferroni correction, none of these differences remained statistically significant (all *p* ≥ .0125). Neither were any significant differences found for *Clarity* (*U* = 1570.5, *p* = .125) or *Relevance* (*U* = 1482, *p* = .052),

For answers authored by health professionals, no significant differences were observed between youth and professional health participants across *Validation* (*U* = 1724.5, *p* = .408), *Relevance* (*U* = 1566, *p* = .120), *Clarity* (*U* = 1571, *p* = .127), and *Utility* (*U = *1554.5, *p* = .110), with *p*-values remaining nonsignificant after Bonferroni correction.

**Figure 3.**
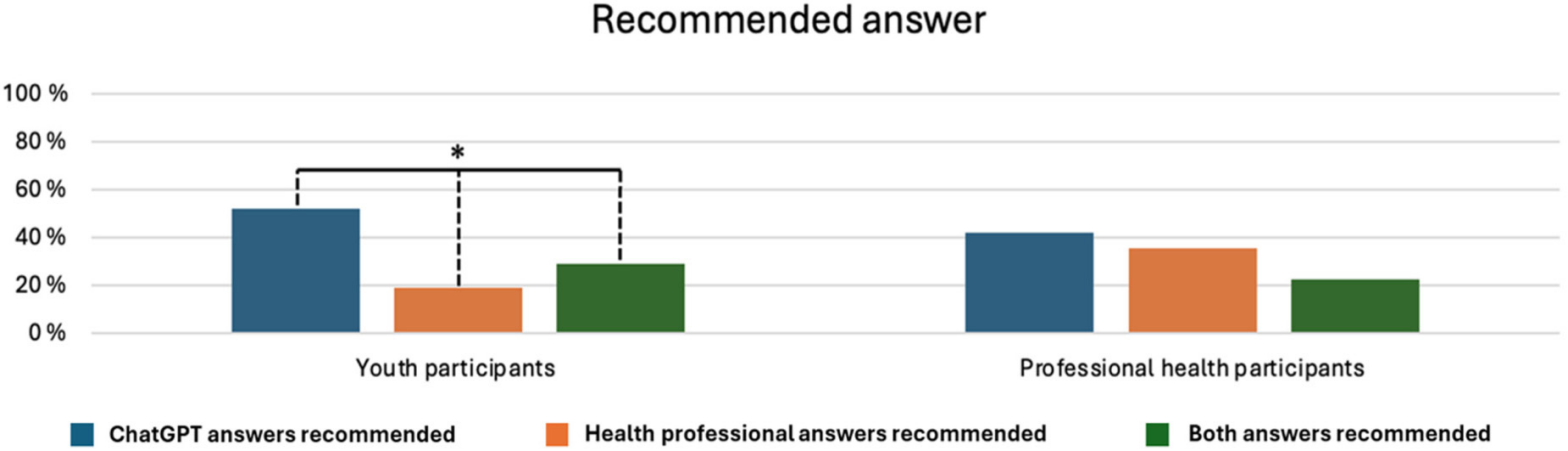
Differences in recommendations among participation groups between answers generated by ChatGPT and those provided by health professionals.

The participants’ preferences for different answers were assessed by asking them to recommend one. Figure 3 shows that nominal differences were discernible between youth and professional participants; however, no statistically significant differences between groups were observed. Compared to 42% of professional health participants, 52% of youth participants recommended one or both ChatGPT answers. Conversely, 19% of youth recommended one or both answers from health professionals, whereas 35% of professional health participants did the same. Lastly, 29% of youth participants and 23% of health professional participants either expressed no preference or recommended the answer from one source for one question and the other source for the other question. A chi-square test showed that these differences were not statistically significant, *χ*^2^(2, *N* = 154) = 4.061, *p* = .131.

#### Perceived differences between answers from ChatGPT and health professionals

This section investigates the differences within the participant groups. First, we analyzed whether and how participants differed in their assessments of answers from ChatGPT and from health professionals (see Figure 2).

Youth participants rated ChatGPT answers significantly higher than those of health professionals for *Relevance* (*z* = –3.284, *p* = .001) and *Utility* (*z* = –3.912, *p* < .001). For *Validation* (*z* = –2.078, *p* = .038) and *Clarity* (*z* = –2.410, *p* = .016), ChatGPT's average ratings were nominally higher than answers provided by health professionals but were not found to be significant at the corrected threshold. A corrected threshold (Bonferroni) was applied to reduce the risk of false positives due to multiple comparisons, implying that only robust group differences remained statistically significant.

Using the same correction in our analysis of professional health participant scores, we found no significant differences between assessments of answers generated by ChatGPT and those authored by professional health participants across *Validation* (*z* = −0.149, *p* = .882), *Relevance* (*z* = −0.878, *p* = .380), *Clarity* (*z* = −1.185, *p* = .236), and *Utility* (*z* = −0.958, *p* = .338).

We also investigated differences within participation groups regarding their recommendations for answers (see Figure 3). For youth participants, the within-group difference was statistically significant, as assessed by a one-sample chi-square test, *χ*^2^ (2, *N* = 123) = 21.415, *p* < .001. No significant within-group difference was found in professional health participants’ recommendations, *χ*^2^(2, *N* = 31) = 1.806, *p* = .405. These findings indicate that youth participants tended to prefer answers from ChatGPT over answers from health professionals. In contrast, professional health participants did not show such a tendency.

### Results from qualitative analysis: Emerging themes reflecting participant perceptions of the strengths and weaknesses of the answers

This section's thematic analysis of participant reports indicates that both participant groups experienced overlap in the positive and negative aspects of AI-generated and professionally written answers.

#### Themes representing positive aspects

Most participants (92% of young people and 94% of health professionals) reported one or more positive aspects of the responses evaluated. These positive aspects were grouped into four main categories: (1) cognitive, (2) relational, (3) relevance, and (4) professional.

**Figure 4.**
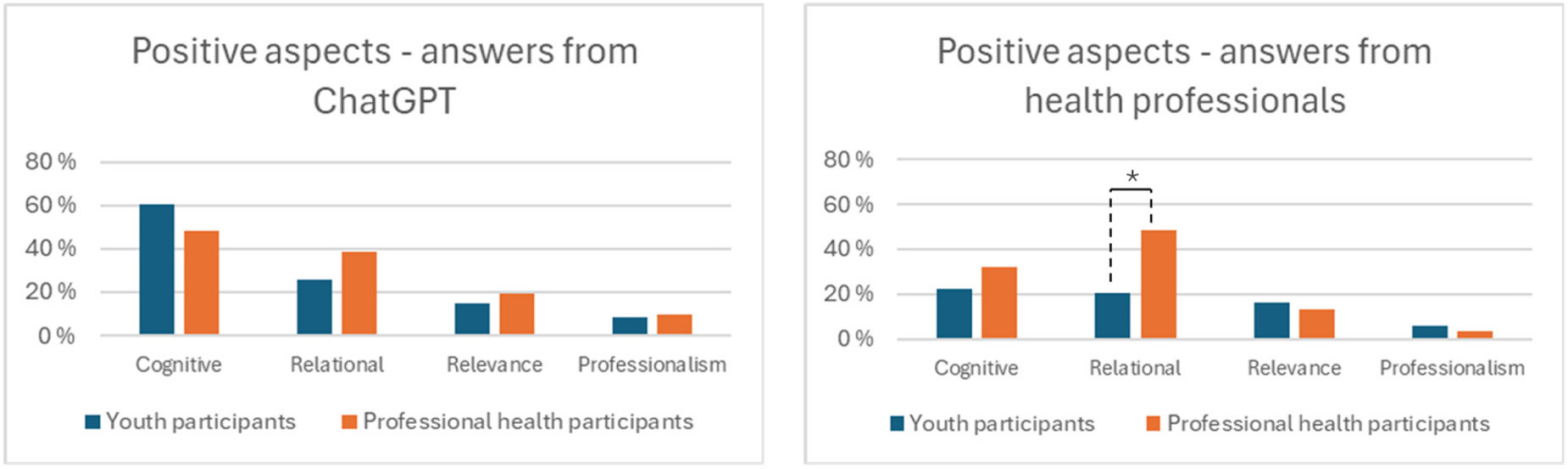
Participant groups reporting positive aspects of the answers generated by ChatGPT compared with those provided by health professionals.

Figure 4 provides a high-level overview of the distribution of categories, while Table 1 provides an overview of the specific positive aspects. The categories are described in detail below.

**Table 1.** Themes reflecting positive aspects of the answers provided by ChatGPT and health professionals.

Category	Themes	Frequencies (%)					
		Youth participants			Professional health participants		
		C	HP	B	C	HP	B
Cognitive aspects	Concrete and actionable advice	43	5	11	26	10	10
	Understandable and easy to process	25	9	7	29	23	0
	Comprehensive and explanatory	20	11	8	6	10	6
Relational aspects	Compassionate and empathic tone	9	11	2	16	29	0
	Sees and supports the person	14	11	7	23	19	6
	Validates or normalizes	5	3	2	26	16	3
	Other relational	5	0	0	3	3	0
Relevance	Adequate answer	3	3	7	6	0	13
	Answer adapted to the person asking	11	13	0	16	13	0
Professional aspects	Professional and credible	7	5	1	6	0	0
	Avoids medical diagnoses	1	1	0	3	3	3
Other	Suggests help services or resources	15	7	2	10	16	0

C: answers from ChatGPT; HP: answers from health professionals; B: answers from both.

We compared participants’ reports on the cognitive, relational, and relevance categories by pairwise chi-square tests. A significantly larger proportion of professional health participants than of young people emphasized the importance of relational aspects in their reflection on the answers provided by health professionals, *χ*^2^(1, *N* = 154) = 10.14, *p* ≤ .001. Otherwise, no significant differences were observed between the groups. The categories are described in detail below.

*Positive cognitive aspects.* In total, 85% of youth and 74% of professional health participants reported positive cognitive aspects in their feedback, particularly regarding the answers generated by ChatGPT. Both participant groups reported valuing concrete answers that contained relevant, actionable advice. Such advice could be communicated through techniques, tips, or examples, as described by the youth participant ID83:

The one to the right [ChatGPT] provides solutions and presents suggestions that may help the person. It also show how you can improve things in your everyday life if the situation were to arise again.

The participants also valued comprehensive, explanatory answers that were rich in detail and facilitated an in-depth understanding or a solution to the problem. Answers should be well-structured, easy to read, and easy to understand. Some emphasized the importance of using simple language or bullet points to make the content easier to grasp, as demonstrated by the youth participant ID26:

The other one is structured systematically with bullet points, which helps the reader get a clearer overview of what can be done. It is much easier to absorb information when you don’t have to search for it. That way, the answer to the question is right in front of you, and you don’t have to look for it every time you read the text.

*Positive relational aspects.* Positive relational aspects of the answers were particularly important for professional health participants, with 81% mentioning them in their feedback, compared to 50% of youth. Nominally, youth participants mentioned relational aspects more for ChatGPT, while health personnel mentioned them more for health professionals’ answers. The participants positively responded to answers that were motivating, friendly, personal, or showed empathy in ways that made them feel seen and heard. For some, feelings of being seen could be linked to levels of detail in the answer, as demonstrated by youth participant ID110:

I thought the answer on the right side was the best. It provided a thorough explanation and conveyed that they cared and genuinely wanted to help. However, it also hinted that if things took a wrong turn, one should seek professional help.

Some of the participants also noted that answers should validate or normalize the topic or state of the person, as expressed by the professional health participant ID18:

It takes the young person seriously and provides concrete tips on managing anxiety. It acknowledges that anxiety is uncomfortable but not dangerous and that it doesn’t have to limit daily life.

*Answer relevance*. The relevance of answers was addressed in feedback from 36% of youth participants and 42% of professional health participants, with similar distributions across both sources for both participant groups. Both groups viewed an answer positively when it was appropriate and addressed the question, as demonstrated by youth participant ID103:

I think the first answer was one where you felt seen, and the person responding tried to understand how the young person was feeling. I think the second answer was a good response with a lot of valuable information.

*Positive professional aspects.* The perception of answers as professional, credible, and trustworthy—written by someone with significant experience—was reported by 15% of youth participants and 16% of professional health participants, indicating similar importance across both groups. The following comment from youth participant ID56 captures this sentiment:

Again, both answers were good, but the one on the right is clearly of higher quality than the one on the left. The left one sounds like something you could read on the internet, while the right one sounds like it comes from an experienced person.

Participants also considered it positive when an answer abstained from discussing or referring to medical diagnoses, as exemplified by professional health participant ID34:

Good support. Clearly states that a diagnosis cannot be made online.

*Other aspects.* Participants valued answers that emphasized the possibility of reaching out to other help services and resources, such as health professionals or teachers, as exemplified by youth participant ID129:

In addition, it includes important points such as seeking professional help when necessary, which shows care for the user's mental health.

#### Themes representing perceived problems with an answer

Participants also reported problems with the answers provided by both ChatGPT and health professionals. Such issues were identified in one or both reports by 49% of youth participants and 84% of professional health participants. The identified concerns have been categorized into the same four categories as the positive aspects discussed in the previous subsection.

**Figure 5.**
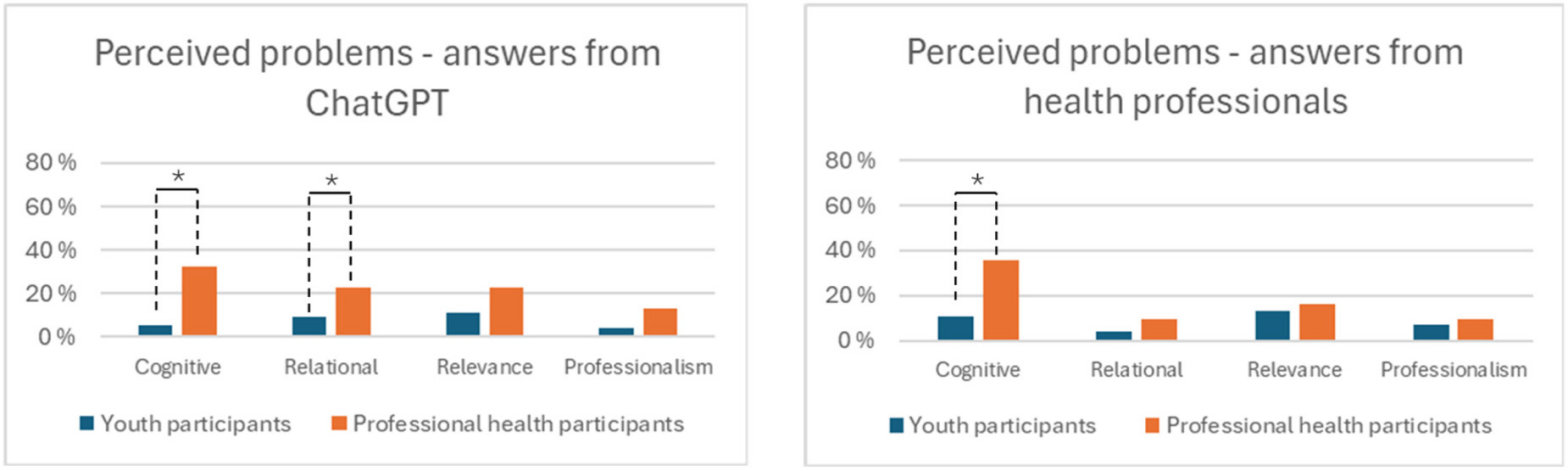
Participant groups reporting problems with of the answers generated by ChatGPT compared with those provided by health professionals.

Figure 5 provides a high-level overview of themes across the four categories, while Table 2 provides an overview of the specific problems reported. The categories are then described in detail.

**Table 2.** Themes reflecting negative aspects of the answers provided by ChatGPT and health professionals.

Category	Theme	Frequencies (%)					
		Youth participants			Professional health participants		
		C	HP	B	C	HP	B
Cognitive aspects of the answer	Too detailed or difficult to process	1	8	2	16	21	3
	Too many advice	0	0	0	10	3	0
	Difficult words and expressions	4	1	0	16	6	0
	Poorly structured text	1	2	0	0	3	0
Relational aspects of the answer	Too factual	9	4	2	23	6	0
	Lack of validation	0	0	0	0	3	0
Relevance of the answer	Too general	11	2	0	13	3	0
	Insufficient advice or answer	1	7	0	0	10	3
	Irrelevant content	0	4	1	10	3	0
Professional aspects of the answer	Unprofessional	2	3	0	0	0	0
	Opinionated	1	2	0	0	6	0
	Concerns medical diagnosis	2	2	1	13	3	6
Other	Other or uncertain	2	1	8	0	0	0

C: answers from ChatGPT; HP: answers from health professionals; B: answers from both.

Chi-square tests showed that significantly more professional health participants emphasized the importance of problems concerning cognitive, *χ*^2^ (1, *N* = 154) = 17.795, *p* ≤ .001, and relational, *χ*^2^(1, *N* = 154) = 4.461, *p* ≤ .05, aspects when reporting on answers from ChatGPT. A similarly significant difference was also found for cognitive aspects related to answers from health professionals, *χ*^2^(1, *N* = 154) = 11.683, *p* ≤ .001. No other significant differences in terms of reported problem categories were observed.

*Problems concerning cognitive aspects.* Problems related to “cognitive aspects” were more frequently reported by professional health participants (61%) than by youth participants (16%), and across responses from both ChatGPT and health professionals. When answers were found to be too comprehensive, too detailed, included too much advice, or were hard to understand due to difficult words, professional health participants were most often negative. A lack of proper structure was also reported as undesirable.

I think answer 1 was way too long. I felt there was too much talking, and I didn’t really get a clear, concrete answer. (ID101, Youth Participant)

*Problems concerning relational aspects.* Problems concerning “relational aspects” refer to instances in which the answers were overly factual, lacking sufficient warmth or empathy, or failed to normalize or validate the issue or the person.

The one on the left was more professional and not as “emotional,” so the person may not have felt seen, depending on the individual. (ID68, Youth Participant)

This concern was reported more often by the professional health participants (32%) than by the youth participants (15%), and it was nominally more often associated with answers from ChatGPT.

*Problems concerning answer relevance.* This category was nominally more often reported by professional health participants (42%) than by youth participants (23%). For those professional health participants, the concerns were nominally more often related to ChatGPT-generated answers, but not to a degree that was significant. Lack of relevance could take the form of answers that did not address the question, provided only superficial information, or lacked concrete advice.

For example, DSM-5/ICD-10 is not relevant information for young people, and mentioning a psychiatrist feels a bit excessive. Both answers contain relevant information, but the warmth is lost in the one on the right. (ID40, Professional Health Participant)

Problems concerning professional aspects. Problems concerning “professional aspects” were associated with answers experienced as patronizing, lacking accuracy, or otherwise inappropriate in the given context. Answers were also perceived negatively when reported to be too opinionated, insistent on a particular perspective, or overly oriented toward suggesting or referring to medical diagnoses.

Answer number two pathologizes the sender and mentions diagnoses, which should not be done. (ID19, Professional Health Participant)

This category was nominally more frequently mentioned by the professional health participants (29%) than by the youth participants (12%), and more of the concerns raised by the professional health participants concerned ChatGPT-generated answers.

## Discussion

By comparing how youth (the primary users) and health professionals (the expert providers) evaluate advisory answers from ChatGPT and from human professionals, our study identifies ChatGPT's perceived strengths and limitations. These findings offer insight into how LLMs can support mental-health communication and advisory services.

Our results indicate that ChatGPT and health professionals both provide answers that are experienced as valuable, relevant, and easy to understand. The participants appreciated concrete, actionable, and well-structured answers with references to additional helplines, features that may enhance the perceived richness of communication. Trustworthiness and empathy also played a key role in a positive reception. Conversely, answers were viewed negatively, or as less rich, when they were unstructured, difficult to understand, overly vague, excessively detailed, generic, or emotionally distant. Notably, our findings align with previous research, showing that answers from ChatGPT are highly rated by both participant groups, though youth participants tend to be more positive.

### Few differences between young people and health professionals

An interesting and somewhat unexpected finding in response to our research questions concerns the similarities between the two groups in their evaluations of answers to mental health questions. Despite clear differences in expertise, developmental stage, and communicative expectations, youth and professional health participants displayed only minor divergences in their ratings. Young people rated ChatGPT's responses nominally higher on *Validation*, *Relevance*, *Clarity*, and *Utility*, but these differences were not significant after Bonferroni corrections. The two groups were equally likely to recommend ChatGPT answers or those from health professionals.

Our qualitative data analysis somewhat reflects these observations, with similar themes identified in both groups and a comparable distribution across these themes. However, several key differences arose: professional health participants emphasized the relational aspects of responses from other health professionals and were more critical in their evaluations, reporting problems related to cognitive (both) and relational aspects of the answer (ChatGPT). They tended to find it problematic if an answer was too comprehensive, used difficult words, or came across as too factual. Their assessment likely reflects the established health communication guidelines on the www.ung.no platform, and ChatGPT may produce answers that are less consistent with these guidelines, encouraging a more critical stance.

The fact that both groups were equally likely to recommend an answer from ChatGPT or a health professional suggests that, despite their more critical stance, professional health participants may have found ChatGPT sufficiently rich for mental health communication. Regardless of their very different backgrounds, young people and health professionals seemed to assess the answers using almost the same criteria. This challenges the expectation that these groups may differ in what they consider helpful. Instead, our results suggest that in the context of text-based interactions, clarity, accessibility, and a sense of being understood are shared standards for what makes mental-health guidance feel useful.

### Determinants of rich communication in text-based mental health

Despite similarities between the two groups, young people rated ChatGPT answers significantly higher than those of health professionals in terms of *Relevance* and *Utility* and generally showed a substantial preference for ChatGPT-generated answers. Professional health participants showed no such preference and rated answers from both sources similarly. This indicates that although youths see ChatGPT as a particularly helpful and relevant source of guidance, professionals do not evaluate it as superior to answers written by humans. In reflecting on their choices, participants applied similar criteria to their evaluations of answers: how clear they were, how relevant they felt, how supportive they seemed, and how professional they appeared. Answers were viewed positively when they were easy to understand, relevant to the person asking, validating, and credible. ChatGPT was often seen as offering clearer, more structured answers, whereas health professionals were more often described as providing responses that felt more personally supportive. This suggests that the two sources offer different strengths rather than one being better overall.

In a text-based and mental-health context, the determinants of “rich” communication may differ from those assumed in classical Media Richness Theory. While traditional richness emphasizes multiple cues and immediate feedback, our findings suggest that, in this context, richness is tied to the cognitive, relational, and professional qualities of the written answer, as well as its relevance.

The inclination among young people toward LLM-generated advice aligns with the extant literature.^35,39^ In line with the Media Richness Theory, young people may perceive ChatGPT's responses as information-rich because they provide relevant, actionable advice in easy-to-understand language and structure, following a format that aligns with their digital communication habits. By reducing perceived ambiguity in health-related inquiries, ChatGPT may enhance clarity and accessibility, which can be crucial for younger audiences, particularly when dealing with emotionally charged or confusing mental-health concerns. Previous research has found that ChatGPT and similar services are particularly effective at improving reading quality by generating well-structured texts^36,48^ and providing detailed, actionable advice that supports perceptions of helpfulness.^17^ In contrast, professional health participants who already possess domain expertise may not experience the same reduction in ambiguity when reading ChatGPT-generated answers. Consequently, they may not exhibit a strong preference for ChatGPT-generated responses over those authored by health professionals.

The qualitative data also indicated that young people found ChatGPT made them feel seen, supported, or validated, a pattern consistent with other studies on LLM-generated responses.^35,39^ Interestingly, ChatGPT's ability to convey care and empathy may be closely linked to the details in its answers, making its messages feel “richer” in a text-based context. A comprehensive answer may suggest that the author has taken the time to provide a thoughtful reply, which could then foster a sense of being cared for. However, a potential tendency to infer care from comprehensive answers could create misplaced trust or overreliance on AI-generated advice.^49^ The latter exemplifies how LLMs can accumulate relational authority and “model power” by appearing knowledgeable, attentive, and emotionally supportive. This is particularly important given that recent reports show that millions now turn to LLMs such as ChatGPT for emotional support and mental-health advice, raising urgent questions about the readiness and safety of these tools.^50^

In contrast, professional health participants demonstrated greater skepticism toward the relational aspect of answers generated by ChatGPT. They noted that ChatGPT tended to be overly factual, potentially reducing an answer's ability to convey care and empathy. It is likely that professional health participants have different expectations for how validation should be delivered by health communication. This assumption is further strengthened by the group's tendency to emphasize positive relational aspects in responses from other health professionals. As Young et al.^17^ demonstrated, the perception of empathy varies by context. When communicating health information, health professionals may expect a nuanced display of validation and empathy, something that LLMs may not be able to provide.^51^

Taken together, the findings imply that the determinants of rich media communication shift in a text-based mental-health support context, especially for young people. This mirrors the idea that media richness is not a fixed attribute but depends on the communication context and the communicative task.^40,41^

### The need for a hybrid model

Our findings may have implications for how LLMs should be integrated into online help services to provide support for youth-facing mental health questions. ChatGPT and similar LLMs may serve as effective supplements that can scale up mental health support, providing clear, structured explanations and lowering informational barriers for many young users in a short time frame. However, the relational and professional limitations of LLMs, as identified by health professionals, underscore the need for careful oversight.

As such, we suggest a hybrid model that would see collaboration between health professionals and LLMs. Given the resource-intensive nature of human advisory services, this human-AI collaboration could scale up and optimize youth advisory services, particularly in mental health.^25^ A hybrid approach could enhance efficiency^52^ or improve response quality^53^ by utilizing LLM-enabled writing and knowledge acquisition. ChatGPT appears to provide information in a way that makes it easy for young people to understand and could be used by health professionals to improve information clarity while preserving their expertise and domain knowledge. However, the concerns raised by the professional health participants about ChatGPT's answers being too comprehensive, using inappropriate diagnostic language, and lacking efficient relational skills highlight the limits of LLMs and reinforce the need for human involvement.

A hybrid model can be justified by the complementary strengths of both actors. LLMs can deliver accessible, consistent, and easy-to-understand advice, while health professionals are critical to ensuring clinical accuracy, safeguarding ethical standards, and contextualizing advice to platform guidelines and the specific situation of the young person in question, particularly in complex cases. This is important because LLM support alone can feel safe, empathic, and informative, but it also involves risks of overreliance and uncritical trust. Therefore, we argue for a hybrid model in which health professionals collaborate with AI systems rather than replace one another.

#### Ethical and practical considerations with hybrid advisory models

While integrating AI into advisory roles presents several advantages, it also raises important ethical and practical challenges. One major concern is ensuring that AI-generated responses remain unbiased and accurately reflect professional standards. Additionally, safeguarding user privacy and data security is critical when handling sensitive information through AI systems. Another issue that must be addressed is accountability, with clear protocols for handling errors or potential harm caused by AI-generated advice. For example, relying on AI-generated responses may result in overly generic or contextually inappropriate advice, potentially leading to user misunderstandings or misinformation.^54^ Overcoming these challenges is crucial for developing a reliable and ethically sound AI–human advisory framework. Future research should focus on refining hybrid models, establishing clear guidelines for the use of AI in mental health support, and evaluating the long-term impact of AI-assisted advisory services**.**

### Limitations and future research

This study has some key limitations. First, the sample size is relatively small. However, given the study's exploratory nature, the sample size is sufficient to indicate differences in participants’ assessments of answers from the two sources. Additionally, no a priori power analysis was conducted for either participant group. Although our youth sample is relatively large, and the professional sample reflects nearly the entire eligible population, possible undersampling may limit our ability to detect smaller effects and reduce the statistical precision of group comparisons. Furthermore, we used a Bonferroni correction to reduce the chance of Type I error, increasing the risk of Type II errors.^55^ It should also be noted that we did not explore subgroup differences (e.g., gender, age, or educational background), an issue that future research should address.

Second, for ethical reasons, we excluded sensitive mental health topics and do not know how participants would have perceived answers on other, more sensitive issues. According to Young et al.,^17^ preferences may depend on the topic.

Third, while youth participants tended to prefer answers from ChatGPT over those from health professionals, this may reflect youths’ desire for more actionable guidance than current Norwegian guidelines permit. Norwegian health professionals who respond to youths’ online questions through services such as ung.no are typically restricted from providing individualized healthcare recommendations, which is defined as “any action that has a preventive, diagnostic, therapeutic, health-preserving, rehabilitative, or nursing and care purpose and is carried out by health personnel.”^56^ In contrast, ChatGPT may have generated answers that resemble in-person healthcare guidance, making them more useful and specific.

Fourth, it is important to note that we have not independently evaluated the usefulness, accuracy, or specificity of these answers beyond the participants’ assessments. However, the health professionals who participated in this study did not identify incorrect statements provided by ChatGPT.

Fifth, the participants evaluated answers to questions posed by other young people. It is reasonable to assume that those who originally posed the questions were in a different personal context than the study participants. Consequently, participants’ perceptions of the answers must be understood in relation to their own experiences rather than the original askers’ situations. Moreover, individual differences may have influenced how participants perceived the answer. Future research could explore whether youth differ in their assessment.

Finally, the study relied on a single LLM (ChatGPT), and all materials were presented in Norwegian. The results might therefore have differed if another model or language had been used. Given these factors and the rapid development of LLMs, further research is needed to ensure that the findings remain generalizable and relevant.

## Conclusion

To our knowledge, this is the first study to use a blind comparison of how youth and health professionals perceive LLM-generated answers versus those authored by health professionals in a mental health context. By comparing ChatGPT's responses with those of professionals, we could assess its usefulness, accuracy, and limitations in providing mental health guidance. From a media richness perspective, adapted to text-based mental-health communication, the findings suggest that young people perceive ChatGPT differently than health professionals and value ChatGPT's ability to provide clear, relevant, and validating answers. These qualities make ChatGPT's responses feel richer to young people, even within the limits of a text-only medium. Health professionals, however, appear to be more critical of ChatGPT's answers, likely due to a mismatch between the answers and their training, as well as the guidelines they are obliged to follow on the advisory platform. A hybrid model that combines the LLM's scale and efficiency with clarity and the judgment and expertise of health professionals may improve youth advisory services. Future research should focus on the long-term impact of these integrations, particularly regarding user trust, service effectiveness, and the ethical implications of deploying AI in sensitive advisory contexts.
